# 720 Intensive Insulin Therapy in the Burn Intensive Care Unit: A Systematic Review of Literature

**DOI:** 10.1093/jbcr/irac012.274

**Published:** 2022-03-23

**Authors:** Jasmine N Peters, Zachary J Collier, Justin Gillenwater, Haig A Yenikomshian

**Affiliations:** Keck School of Medicine, Glendale, California; Keck School of Medicine, University of Southern California, Los Angeles, California; USC/LAC+USC Medical Center, Los Angeles, California; USC/LAC+USC Medical Center, Los Angeles, California

## Abstract

**Introduction:**

The significant burden of burn-related morbidity and mortality is partly due to the complex pathophysiological derangements that occur in the acute post-burn period. Critical care literature has pushed for tighter glycemic control, but these studies often use heterogenous groups of medical and surgical patients. Furthermore, some of these studies present conflicting evidence of whether there is true mortality benefit. Providers must balance the risks associated with hyperglycemia such as infection, inflammation, and pour wound healing against the risks associated with severe hypoglycemia, most notably coma and death. This study aims to review the literature on outcomes in tight glucose control regimens (glucose < 150mg/dL) in the burn ICU population to help guide further research and practice guidelines.

**Methods:**

A systematic review of literature utilizing PubMed was conducted for any article published at any time. Searches used the AND function to identify articles with a burn term (*burn injury* OR *burn care* OR *burn ICU* OR *burn* OR *thermal injury* OR *burned*) and a glucose control related term (*glucose control* OR *glycemic control OR glucose management* OR *insulin* OR *metformin* OR *glipizide*). Inclusion criteria were English studies that describe intensive care unit (ICU) glucose management in adult burn patients. Exclusion criteria were involvement of children, animals, and settings outside of the ICU. Case reports, editorials, and position pieces were also excluded.

**Results:**

The search identified 2,154 articles. Full text review of 61 articles identified 8 that met inclusion criteria. Two randomized control trials, 3 retrospective case-control studies, 2 retrospective cohort studies, and 1 systematic literature review. Only 1 study showed mortality benefit of tighter glucose control (< 150 mg/dL) compared to controls (< 200 mg/dL), while 3 studies showed no difference in mortality between cases and controls. Three studies demonstrated a reduction in infectious complications including sepsis, pneumonia, urinary tract infection, and bacteremia. Nearly all studies (6/8) showed increased rates of hypoglycemia with tight control, but very few instances of adverse sequela following hypoglycemia were noted.

**Conclusions:**

Like the broader critical care population, tighter glucose control may be beneficial to burn patients but with some variability. Balancing the complications of hypoglycemia with hyperglycemia continues to be a challenge with no clear guidelines. Further research in a burn specific population would help create a safe and effective treatment algorithm which could be adapted widely.

